# Genome Sequence of *Thermus thermophilus* ATCC 33923, a Thermostable Trehalose-Producing Strain

**DOI:** 10.1128/genomeA.00493-13

**Published:** 2013-07-25

**Authors:** Ling Jiang, Ming Lin, Xiaotong Li, Huaiyan Cui, Xian Xu, Shuang Li, He Huang

**Affiliations:** College of Food Science and Light Industry, Nanjing University of Technology, Nanjing, Chinaa; College of Biotechnology and Pharmaceutical Engineering, State Key Laboratory of Materials-Oriented Chemical Engineering, Nanjing University of Technology, Nanjing, Chinab

## Abstract

*Thermus thermophilus* ATCC 33923 contains a thermostable enzyme that can efficiently catalyze the conversion of maltose into trehalose. Here we report a 2.15-Mb assembly of its genome sequence and other useful information, including the coding sequences (CDS) responsible for biological processes such as DNA replication, DNA repair, and RNA maturation.

## GENOME ANNOUNCEMENT

Trehalose (α-d-glucopyranosyl-1,1-α-d-glucopyranoside) is a naturally occurring nonreducing disaccharide that is widely spread throughout mycobacteria, bacteria, archaea, yeast, fungi, and higher orders of the plant and insect kingdom (1). This simple disaccharide can serve as an energy reservoir and a protectant from various physical and chemical stresses, as well as an additive to food, cosmetics, and medicinal and biological reagents (2). The wide range of applications of trehalose has increased the interest of many researchers into the development of economically feasible production systems. Trehalose synthase (TSase) (EC 5.4.99.16) employs an inexpensive substrate, maltose, and allows one-step formation of trehalose by an intramolecular rearrangement of the α-1,4-linkage of maltose to the α-1,1-linkage of trehalose. This pathway is thought to be the most promising process for industrial manufacture of trehalose (3).

Up to now, TSases were found mainly in bacteria (4), and only TSase from the *Thermus* strain was characterized as a thermostable enzyme (5). The intrinsic stability of this thermostable enzyme and its resistance to denaturing physical and chemical factors are considerable advantages in industrial processes. The trehalose produced by *Thermus thermophilus* strain ATCC 33923 showed its optimal activity levels at 70°C, and the maximum yield was up to 72% for 24 h of the reaction, which is higher than most of the previously published results (6, 7). Therefore, investigation of the genetic information and characteristics of *T. thermophilus* ATCC 33923 is desired to further elucidate this mechanism. Knowledge of the genome sequence and bioinformatics will be of great help in this regard. In addition, genome-scale analysis has proven useful for metabolic engineering applications (8).

Here we present the draft genome sequence of strain *T. thermophilus* ATCC 33923, obtained using an Illumina Hiseq 2000 system, which was performed by Shanghai Majorbio Bio Pharm Technology Co., Ltd. The reads were assembled with Velvet (9), and the sequence was annotated using the RAST annotation server (10). A library containing 300-bp inserts was constructed. Sequencing was performed based on the paired-end strategy of 101-bp reads to produce 541 Mb of filtered sequences, representing a 252.03-fold coverage of the genome. The sequence of *T. thermophilus* ATCC 33923 is 2,147,217 bases with a G+C content of 69.41%, which was assembled into 117 contigs and 94 scaffolds. It contains 2,270 open reading frames (ORFs), 46 tRNA genes, and 2 rRNA genes identified by Glimmer 3.02 (11), Genemark (12), tRNAscan-SE (13), and RNAmmer (14).

According to the genomic analysis, strain *T. thermophilus* ATCC 33923 may have a powerful resistance to physical and chemical agents, as there were 4 ORFs relate to DNA repair and 13 ORFs related to the thermostable protein. Additionally, the vitamin B_12_ and carotenoid biosynthesis enzymes were also annotated in the *T. thermophilus* ATCC 33923 genome sequence. Further studies will be performed to confirm their functions, and a complete genome sequence will be included in the future to reveal the unique molecular characteristics of strain *T. thermophilus* ATCC 33923.

### Nucleotide sequence accession numbers.

This whole-genome shotgun project has been deposited at DDBJ/EMBL/GenBank under accession number AQOS00000000. The version described in this paper is the first version, with accession number AQOS01000000.
